# Higher-Order Aberrations of Topography-Guided LASIK and Wavefront-Optimized LASIK in High- and Low-Myopic Eyes: A Non-Randomized Controlled Trial

**DOI:** 10.3390/jpm13030399

**Published:** 2023-02-24

**Authors:** Elsa Lin-Chin Mai, Chao-Kai Chang, Chia-Yi Lee, Ie-Bin Lian, Chen-Cheng Chao

**Affiliations:** 1 Department of Optometry, MacKay Junior College of Medicine, Nursing, and Management, Taipei 11260, Taiwan; 2 Department of Ophthalmology, Far Eastern Memorial Hospital, Taipei 220216, Taiwan; 3 Department of Optometry, Yuanpei University of Medical Technology, Hsinchu 30015, Taiwan; 4 Nobel Eye Institute, Taipei 100008, Taiwan; 5 Department of Optometry, Da-Yeh University, Changhua 515006, Taiwan; 6 Institute of Medicine, Chung Shan Medical University, Taichung 402306, Taiwan; 7 Department of Ophthalmology, Jen-Ai Hospital Dali Branch, Taichung 412224, Taiwan; 8 Institute of Statistical and Information Science, National Changhua University of Education, Chunghua 50007, Taiwan

**Keywords:** laser in situ keratomileusis, topography-guided, wavefront-optimized, high myopia, higher-order aberration

## Abstract

We aimed to investigate high-order aberration (HOA) change between topography-guided (TG) and wavefront-optimized (WFO) laser in situ keratomileusis (LASIK) in patients with different degrees of myopia. A non-randomized clinical trial was conducted, in which 40 eyes of 20 patients aged 20–50 years old were included. Participants received TG-LASIK in one eye and WFO-LASIK on the alternate eye. Corneal topography and HOAs including coma, trefoil, spherical aberration (SA), and contrast sensitivity (CS) were collected. Moreover, a quality of vision (QoV) questionnaire was completed by each participant. Non-parametric tests were used to infer the difference in HOAs and CS between the TG-LASIK and WFO-LASIK groups, and subgroup analyses stratified by myopia degree were performed. The high-myopia patients with TG-LASIK showed more coma and SA compared to low-myopia individuals (all 95% CI lower limits > 0), and subjects who received WFO-LASIK exhibited more SA in high-myopia status (both 95% CI lower limits > 0). The TG-LASIK group showed lower postoperative trefoil compared to the WFO-LASIK group in the high-myopia population (mean difference: −0.1267, 95% CI: −0.24 to −0.01). The TG-LASIK group yielded less surgically induced haze, better clarity at night, and better total quality scores (all *p* < 0.05). In conclusion, TG-LASIK might yield less postoperative trefoil in high-myopia patients and higher QoV in the general population compared to the WFO-LASIK procedure.

## 1. Introduction

Laser in situ keratomileusis (LASIK) has been established as an effective treatment for refractive errors such as myopia, hyperopia, and astigmatism in recent decades [1]. In a previous study, conventional LASIK was noted to produce high-order aberrations (HOA) after surgery, which cause post-surgical symptoms such as glare, halos, haze, light sensitivity, and loss of contrast sensitivity, which can influence the visual quality [2].

The field of refractive surgery has since shifted to custom correction tailored to the patient’s HOA refractive error, such as wavefront-optimized (WFO) LASIK [3]. However, as these corneal refractive surgery patients age, cataract surgery and intraocular lens calculation become a problem, as the lens aberration must be factored onto the cornea with WFG-LASIK. Changing to an aspherical intraocular lens would probably worsen the spherical aberration (SA) of the patient [4]; therefore, the refractive surgery community has returned to surface treatment, whereby customization occurs on the anterior surface of the cornea. Topography-guided (TG) LASIK customizes the laser treatment to the unique surface condition of the patient, with the target of annihilating HOA [5].

A review of the literature comparing the performance of different LASIK profiles reveals that most patients in these studies have low- or middle-level of myopia [6,7,8,9]. Taiwan’s population has nearly the highest myopia rate in the whole world and, in terms of severity, has the most severe high myopia, i.e., more than 6.0 diopters (D), with up to 22% suffering from this condition [10,11]. With the high amount of myopia, different HOA amounts may be yielded with different LASIK types, such as WFO-LASIK and TG-LASIK; however, this concept requires further study to validate the possibility.

Accordingly, the aim of our study is to analyze and compare outcomes between TG-LASIK and WFO-LASIK in terms of visual performance and the change in different types of HOA, including coma, trefoil, and SA, in patients with high myopia. We also administered a subjective quality of vision (QoV) questionnaire to confirm whether the alleged superiority of TG-LASIK has translated to real-life scenarios and is noticeable to patients.

## 2. Materials and Methods

### 2.1. Patients

The inclusion criteria for this study were as follows: age between 20 and 50 years old, corrected distance visual acuity (CDVA) of both eyes reaching a 0.1 logarithm of the minimum angle of resolution (log MAR), and eyes wit stable refractive errors of myopia and astigmatism within the last 6 months. The exclusion criteria were as follows: cataracts; corneal opacities or irregularities; dry eye (Schirmer’s test, I ≤ 5 mm); amblyopia; coexisting ocular pathologies; glaucoma; non-dilating pupils; history of intraocular surgery, laser therapy, or retinopathy; optic nerve or macular diseases; estimated postoperative cornea residual stromal thickness less than 250 μm; pregnancy or under lactation; uncontrolled diabetic mellitus or systemic immune disease; or refusal or inability to maintain follow-up. This study included 20 patients and 40 eyes, among which 20 eyes received TG ablation and the other contralateral 20 eyes received WFO ablation.

### 2.2. Surgical Technique and Preoperative Assessment

All LASIK surgeries were performed by one of two experienced surgeons (C.K.C. and E.L.C.M.) using an identical technique. In all the operated eyes, a 9.0 mm diameter flap was created using a femtosecond laser (WaveLight FS200, Alcon, Fort Worth, TX, USA) with a hinge on the superior and flap the thickness set to 110 μm; refractive laser ablation was performed utilizing an excimer laser WaveLight EX500 with Contoura Vision software input (both Alcon Laboratories, Fort Worth, TX, USA) in all cases. We designated a Contoura-based TG ablation profile to the dominant eye and a WFO ablation profile to the non-dominant eye in all patients. The treatment target was based on manifest refraction with adjustment using Alcon’s nomogram of LASIK, with both eyes corrected to emmetropia. In the TG group, adjusted manifest refractions were used, which were based on corneal topography images obtained from a WaveLight Topolyzer VARIO device and then transferred to Contoura Vision software to correct the HOA; at least three consistent images were acquired for all eyes according to the manufacturer’s protocol.

### 2.3. Ophthalmic Examinations and Postoperative Assessment

The patients were examined preoperatively, as well as on post-op day 1, the 1st week, 1st month, and 3rd month after surgery. During each visit, we performed a thorough ophthalmologic examination that included tests for UCVA and CDVA, manifest refraction, biomicroscopy, and pneumotonometry. Fundus examination and cycloplegic refraction were performed before surgery. Ocular dominance was evaluated preoperatively by the hole-in-the-card method described in the literature [12]. The following four types of postoperative measurements were used to compare the performance between TG and WFO ablation: (i) ophthalmic examinations, including uncorrected distance visual acuity (UDVA) and CDVA; (ii) cornea wavefront examination, including coma, trefoil, and SA; (3) contrast sensitivity (CS), including 3 cycles per degree (CPD), 6 CPD, 12 CPD, and 18 CPD with glare off and on under photopic and mesopic lighting conditions; and (iv) a QoV questionnaire, including a score of 11 questions and mean total score. Corneal topography and corneal wavefront analyses were performed preoperatively and during the 1st week, 1st month, and 3rd month postoperative visits with a Placido disc system (WaveLight Allegro Topolyzer Vario, Alcon, Fort Worth, TX, USA). Higher-order aberrations were assessed, including the RMS errors of coma (Z 31), trefoil (Z 33), and spherical aberration (Z 40). CS was measured preoperatively and during the 1st week, 1st month, and 3rd month postoperative visits using Vector Vision CSV-1000 (Greenville, OH, USA) chart. All subjects were tested at the recommended distance of 8 feet. CSV-1000 consists of a series of circular achromatic sine-wave patches 1.5 inch in diameter and comprising four rows, each corresponding to one of four spatial frequencies, i.e., 3, 6, 12, or 18 CPD. The QoV questionnaire was administered during the 1st week, 1st month, and 3rd month postoperative visits. Subjective QoV was evaluated using an 11-item questionnaire adopted from a previous study [13]. Our questionnaire contained multiple subscales, including glare at night and glare during the day; haze, halos, and clarity at night; clarity during the day; problems with dry eyes; severity of dry eyes; gritty or sandy feelings; fluctuating vision; and double vision or ghost images. The item scores ranged from 0 to 10, and higher scores on the questionnaire indicated more difficulty in achieving specific visual tasks and subsequent poor QoV. Rasch scaling was used to convert the logistic scale to a linear scale ranging from 0 to 100, with higher scores indicating poorer QoV in the patients.

### 2.4. Statistical Analysis 

Rasch analysis was used to evaluate the QoV questionnaire and determine whether all items measured a single underlying construct [14]. The raw ordinal scores were then converted to interval scores, which were used in parametric statistical tests. We also used Rasch analysis to assess item hierarchy (items were ordered from least to most difficult) and person separation statistics (distinction between groups of participants based on the extent of the underlying construct). The person separation index was set at 2.0. The mean square outfit statistics of each questionnaire item were set as 0.80 to 1.20. We transferred items that fit the Rasch model from the ordinal data to numerical data ranging from 0 to 100. Questionnaire data were analyzed using WINSTEPS Version 4.8.2. Other statistical tests included the Kruskal–Wallis rank test for continuous data, Pearson’s chi-squared test for ordinal data, and Wilcoxon matched-pair signed rank test for matched continuous data. Under the study design, all factors were matched between each pair of TG eye and WFO eye, except for the level of myopia, in which high myopia was defined by a cutoff of more than −6.0D spherical equivalent (SE). We drew a forest plot to show the mean difference of HOAs between the TG-LASIK and WFO-LASIK groups, as well as the low-myopia population and high-myopia population, with a 95% confidence interval (CI). Statistical significance was set at *p* < 0.05.

## 3. Results

### 3.1. Demographic Data and Visual Outcome

Table 1 presents preoperative demographic data and postoperative data. Because of the contralateral eye design of the study, there were no significant differences in gender, CDVA, manifest refraction, CS, or corneal HOA before surgery among the study population (Table 1). In the two stratified groups, neither the refractive spherical nor cylinder were found to have significant differences. The postoperative visual acuity UDVA, CDVA, and SE between the TG-LASIK and WFO-LASIK groups were not significantly different (Table 2).

### 3.2. HOAs and CS Outcome

**Figure 1:**
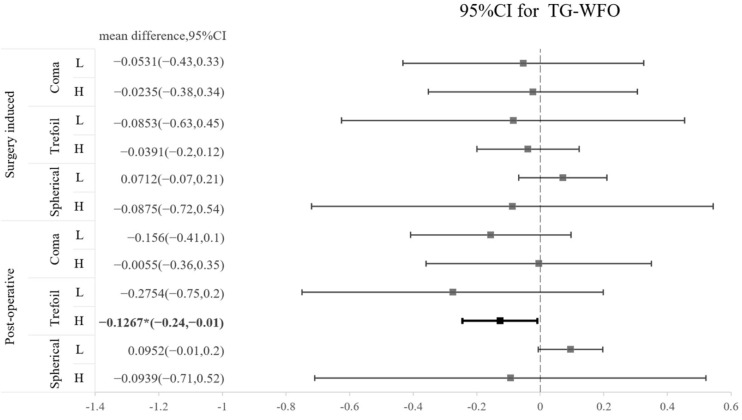
Forest plot of the standardized mean difference in surgically induced HOA or postoperative HOA between TG and WFO on coma, trefoil, and spherical aberration with 95% confidence intervals stratified by level of myopia (L: low; H: high).

The SA and coma in both groups were significantly increased after operation, whereas trefoil aberration did not show a significant increase after operation in either type of surgery (Table 3). A comparison of the three types of HOA between TG-LASIK and WFO-LASIK is presented as a forest plot in Figure 1. We found that trefoil was marginally lower by TG-LASIK ablation in high-myopia patients than in the WFO-LASIK ablation group, with a mean difference of −0.1267 (CI −0.24 to −0.01); however, this result was not observed in the surgically induced trefoil (Figure 1). The CS 3 months after the LASIK procedure did not reveal a significant difference between the TG-LASIK and WFO-LASIK groups (all *p* > 0.05) (Table 4).

### 3.3. High-Myopia vs. Low-Myopia Analysis for HOAs and CS

**Figure 2:**
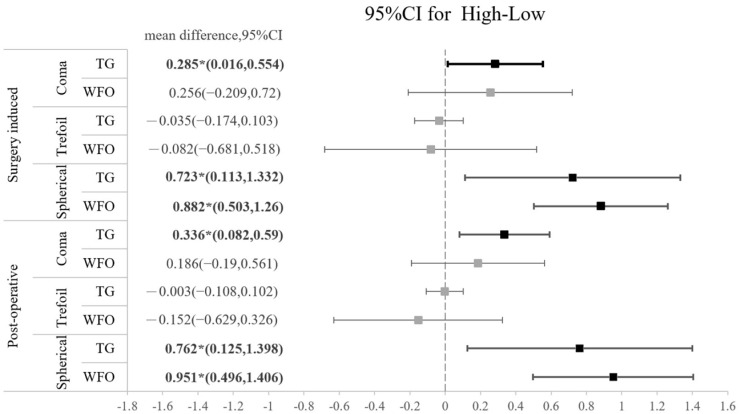
Forest plot of the standardized mean difference of surgically induced HOA or postoperative HOA between high myopia and low myopia on coma, trefoil, and spherical aberration with 95% confidence intervals stratified by TG vs. WFO.

**Figure 3:**
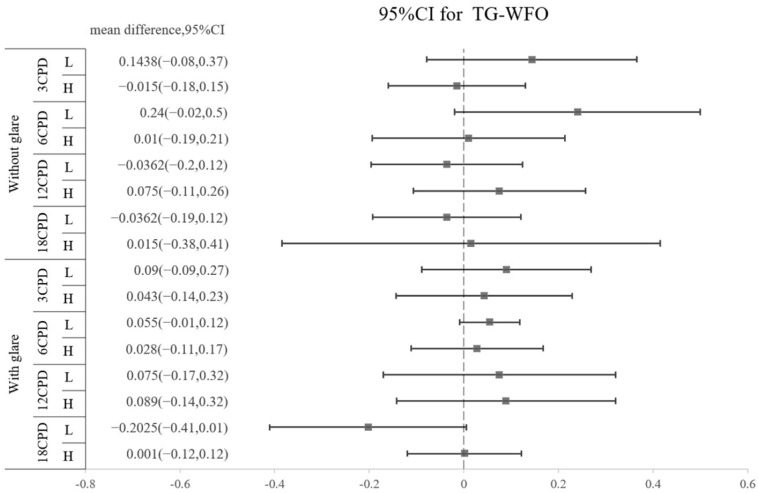
Difference in postoperative contrast sensitivity between TG and WFO stratified by cycles per degree (3, 6, 12, and 18 CPD), glare (off and on) and level of myopia (high and low).

The amounts of all HOAs, including the surgically induced and postoperative types, are shown in Table 5. No significant difference was found regarding the amount of any HOA between the TG-LASIK and WFO-LASIK groups (all *p* > 0.05). As shown in Figure 2, high-myopia patients had higher SA than low-myopia patients, regardless of the TG or WFO ablation method. The surgically induced mean difference for SA is 0.8235 (95% CI: 0.518 to 1.216) for TG and 0.838 (95% CI: 0.473 to 1.204) for WFO in the surgically induced group. In the postoperative group, the SA standardized mean difference is 0.7955 (95% CI: 0.329 to 1.358) in TG and 0.996 (95% CI: 0.508 to 1.377) in WFO, and both are statistically significant. However, the number of refractive corrections does not affect trefoil aberration; the mean difference for the high-myopia minus low-myopia group is −0.035 (95% CI: −0.174 to 0.103) for the TG-LASIK group with surgically induced trefoil and −0.082 (95% CI: −0.681 to 0.518) for the WFO group. Similar findings were found in postoperative trefoil (mean difference: −0.003; 95% CI: −0.108 to 0.102). For surgically induced coma aberration, an intriguing trend was observed in the high-myopic patients that marginally increased after TG-LASIK and decreased after WFO-LASIK, although this was not statistically significant, as shown in Table 5. Moreover, the CS did not illustrate any difference between the TG-LASIK and WFO-LASIK groups in any CPDs, whether with low or high myopia (Figure 3).

### 3.4. Subjective Quality of Vision (QoV) Result

Table 6 presents the subjective optical quality data obtained from the questionnaire responses. For vision tasks, patients with TG-LASIK ablation were more satisfied with respect to haze (35.3 ± 6.7 vs. 37.8 ± 5.4, *p* = 0.0215), clarity at night (34.6 ± 9.1 vs. 38.4 ± 6.4, *p* = 0.0386), and total scores (35.8 ± 6.1 vs. 37.3 ± 4.6, *p* = 0.0225) than patients who received WFO-LASIK ablation. 

## 4. Discussion

The current study shows similar results with respect to UDVA, CDVA, and SE between the TG-LASIK group and the WFO-LASIK group. TG-LASIK induced less trefoil than WFO-LASIK in the high-myopia group, while other HOAs were similar between the two groups. Moreover, the TG-LASIK group reported higher postoperative satisfaction and QoV.

The overall rating of TG-LASIK on the postoperative adverse symptom questionnaire was more satisfactory than that of WFO-LASIK, particularly for the items “haze” and “clarity at night”. A previous study showed that postoperative higher-order aberrations could negatively affect QoV and may cause visual disturbance, especially with coma and spherical aberration [15]. This is in line with our results indicating less postoperative and surgically induced trefoil from TG-LASIK. 

The postoperative SA in both groups significantly increased (*p* = 0.0001), and TG-LASIK yielded more surgically induced comas in the high-myopia group. The induction of SA could be due to the ablation-induced change in corneal asphericity and may be positively correlated with the amount of refraction correction. The induction of coma aberration may also result from subclinical decentrations or asymmetry from wound healing [16]. In a study by Alio, patients with high myopia who received WFO-LASIK had spherical aberration that increased from 0.22 ± 0.11 μm to 0.57 ± 0.15 μm, as well as coma that increased from 0.22 ± 0.15 μm to 0.58 ± 0.28 μm [17]. In another retrospective case series, significant induction of coma aberration and SA after 3 months were also observed in high-myopia patients after refractive correction (coma aberration: 0.21 ± 0.17 μm to 0.58 ± 0.37 μm; and SA: 0.22 ± 0.09 μm to 0.59 ± 0.14 μm) [18], and a similar trend was observed in high-astigmatism patients receiving LASIK treatment [19]. The magnitude of induced SA may be correlated with the amount of achieved correction of SE and positively correlated with corneal asphericity [20].

The postoperative HOA of trefoil was noticeably lower following TG-LASIK in high-myopia patients than following WFO-LASIK; however, this result was not observed in terms of surgically induced difference. We think this may be a relatively novel finding to compare the postoperative HOA with different degrees of myopia between TG-LASIK and WFO-LASIK. Although the surgically induced difference in trefoil between the TG-LASIK and WFO-LASIK groups seems to be more prominent in the low-myopia group, statistical analysis revealed that TG-LASIK exhibited no superiority in terms of surgically induced trefoil reduction in the low-myopia population. The reason for these conflicting results may be the prominent standard deviation of surgically induced trefoil in the WFO-LASIK population, which can cause insignificant differences between the two groups. Furthermore, a trend of better TG results can be observed, with a mean difference leaning towards less HOA in coma and trefoil. The superiority of TG-LASIK in trefoil was also noted in study by Kim et al. (2019) in which the ocular trefoil was reported to be significantly lower after topography-guided ablation than at baseline [7]. However, in the same study, the magnitude of surgically induced ocular coma and SA did not differ significantly between the two groups. We think TG might induce less trefoil aberration in highly myopic patients, but further studies with larger samples are needed to clarify this concept. A major study carried out in 2001 showed that in virgin, unoperated eyes, the first surface of the cornea and internal optics partially compensate for each other’s aberrations to produce an improved retinal image [4]. Thus, it is not always beneficial to remove corneal aberrations. Because there is very little irregularity in virgin eyes, we look specifically into those high myopic patients with more aberration over the cornea. High-myopia patients may benefit from the use of TG-LASIK with better HOA and postoperative QoV, while most patients have regular corneas that are easily treated with WFO-LASIK.

This study is subject to several limitations. First, the number of patients included was limited, with less than 30 eyes in each group, which may have contributed to statistical bias; however, the contralateral eye design with pairwise data comparison might overcomes this shortcoming. Second, the design of the ablation profile was based on ocular dominance but not randomized, which may have caused bias in estimating subjective questionnaire outcomes. Moreover, different wavefront programs may have different effects on HOAs, some of which may have showed better HOA reductions than the TG program. Nevertheless, we cannot compare the TG program of our device to the wavefront program in other devices since we have only one excimer laser device in our institution.

## 5. Conclusions

In conclusion, TG-LASIK might contribute to less trefoil in high-myopia patients compared to WFO-LASIK. Our study shows that high-myopia patients correlate exhibit numerically higher SA and coma, regardless of TG or WFO-LASIK profiles, as compared to low-myopia patients. Moreover, our subjective QoV questionnaire shows higher patient satisfaction in the TG-LASIK group.

## Figures and Tables

**Table 1:** Preoperative demographics, visual acuity, contrast sensitivity, and corneal wavefront aberration between the two groups.

Parameter	TG-LASIK (20 Eyes)	WFO-LASIK (20 Eyes)	*p* Value
Age	32.7 ± 7.8	32.7 ± 7.8	1.0000
Sex (male) (%)	7 (33.3%)	7 (33.3%)	1.0000
Pre-op CDVA (logMAR)	0.01 ± 0.02	0.02 ± 0.04	0.7886
Sphere (D)	−5.70 ± 2.26	−5.89 ± 2.08	0.7148
Cylinder (D)	−0.81 ± 0.61	−0.98 ± 0.90	0.6652
logMesopic CS			
Without glare			
3CPD	1.67 ± 0.22	1.66 ± 0.25	0.5740
6CPD	1.86 ± 0.26	1.88 ± 0.19	0.9780
12CPD	1.55 ± 0.29	1.65 ± 0.29	0.4920
18CPD	1.10 ± 0.27	1.16 ± 0.18	0.6383
With glare			
3CPD	1.62 ± 0.28	1.64 ± 0.29	0.9228
6CPD	1.84 ± 0.27	2.63 ± 3.39	0.4161
12CPD	1.56 ± 0.31	1.59 ± 0.27	0.7422
18CPD	1.08 ± 0.28	1.17 ± 0.27	0.2767
RMS (μm)			
Coma	0.45 ± 0.16	0.48 ± 0.27	0.7868
Trefoil	0.27 ± 0.15	0.38 ± 0.20	0.0699
Spherical	0.56 ± 0.23	0.56 ± 0.24	1.0000

CDVA: corrected distance visual acuity; D: diopter; SE: spherical equivalent; TG: topography-guided; WFO: wavefront-optimized; LASIK: laser in situ keratomileusis; CS: contrast sensitivity; RMS: root mean square.

**Table 2:** Postoperative visual acuity and spherical equivalent at 3 months.

Parameter	TG-LASIK (20 Eyes)	WFO-LASIK (20 Eyes)	*p* Value
UDVA(logMAR)	−0.03 ± 0.07	−0.01 ± 0.10	0.4634
CDVA(logMAR)	−0.05 ± 0.08	−0.04 ± 0.07	0.7439
SE(D)	−0.05 ± 0.29	−0.09 ± 0.28	0.6350

UCVA: uncorrected distance visual acuity; CDVA: corrected distance visual acuity; SE: spherical equivalent; TG: topography-guided; WFO: wavefront-optimized; LASIK: laser in situ keratomileusis.

**Table 3:** Postoperative change in corneal wavefront aberration for the TG and WFO-LASIK groups after 3 months.

RMS (μm)	Preoperative	Postoperative	*p* Value
**TG-LASIK (20 eyes)**			
Coma	0.45 ± 0.16	0.70 ± 0.32	0.0028 *
Trefoil	0.27 ± 0.15	0.22 ± 0.10	0.2180
Spherical	0.56 ± 0.23	1.88 ± 0.72	0.0001 *
**WFO-LASIK (20 eyes)**			
Coma	0.48 ± 0.27	0.70 ± 0.39	0.0315 *
Trefoil	0.38 ± 0.20	0.41 ± 0.39	0.3317
Spherical	0.56 ± 0.24	1.94 ± 0.64	0.0001 *

TG: topography-guided; WFO: wavefront-optimized; LASIK: laser in situ keratomileusis. Wilcoxon matched-pairs signed rank test: * *p* < 0.05.

**Table 4:** Preoperative and postoperative contrast sensitivity between TG-LASIK groups after 3 months.

Mesopic CS (log Units)	Preoperative	Postoperative	*p* Value *
**TG-LASIK (20 Eyes)**			
Without glare			
3CPD	1.67 ± 0.22	1.68 ± 0.21	0.7051
6CPD	1.86 ± 0.26	1.91 ± 0.25	0.3018
12CPD	1.55 ± 0.29	1.56 ± 0.23	0.7592
18CPD	1.10 ± 0.27	1.17 ± 0.33	0.5616
With glare			
3CPD	1.62 ± 0.28	1.71 ± 0.23	0.1831
6CPD	1.84 ± 0.27	1.86 ± 0.13	0.8509
12CPD	1.56 ± 0.31	1.54 ± 0.25	0.9250
18CPD	1.08 ± 0.28	1.06 ± 0.23	0.9102
**WFO-LASIK (20 eyes)**			
Without glare			
3CPD	1.66 ± 0.25	1.62 ± 0.18	0.7758
6CPD	1.88 ± 0.19	1.81 ± 0.26	0.2930
12CPD	1.65 ± 0.29	1.52 ± 0.18	0.0841
18CPD	1.16 ± 0.18	1.18 ± 0.31	0.8344
With glare			
3CPD	1.64 ± 0.29	1.62 ± 0.20	0.9395
6CPD	2.63 ± 3.39	1.81 ± 0.18	0.1994
12CPD	1.59 ± 0.27	1.45 ± 0.20	0.0753
18CPD	1.17 ± 0.27	1.15 ± 0.25	0.8800

CS: contrast sensitivity; TG: topography-guided; WFO: wavefront-optimized; LASIK: laser in situ keratomileusis; CPD: cycles per degree. Wilcoxon matched-pairs signed rank test: * *p* < 0.05.

**Table 5:** Comparison of clinical characteristics between high- and low-myopia groups by TG and WFO ablation after 3 months.

Parameters	TG-LASIK	WFO-LASIK	*p* Value *
**Low myopia**	**(10 eyes)**	**(8 eyes)**	
Pre-op SE (D)	−4.31 ± 0.86	−4.36 ± 1.07	0.8590
Surgically induced (μm)			
Coma	0.16 ± 0.34	0.11 ± 0.47	0.9292
Trefoil	−0.06 ± 0.16	0.08 ± 0.69	0.5940
Spherical	0.96 ± 0.31	0.87 ± 0.39	0.4239
Postoperative (μm)			
Coma	0.57 ± 0.27	0.65 ± 0.33	0.4772
Trefoil	0.22 ± 0.08	0.51 ± 0.55	0.0914
Spherical	1.52 ± 0.30	1.38 ± 0.36	0.2863
**High myopia**	**(10 eyes)**	**(12 eyes)**	
Pre-op SE	−7.78 ± 1.53	−7.69 ± 1.24	0.7920
Surgically induced (μm)			
Coma	0.35 ± 0.31	−0.19 ± 1.55	0.2914
Trefoil	−0.04 ± 0.18	0.00 ± 0.31	0.8691
Spherical	1.67 ± 0.81	1.72 ± 0.32	0.3913
Postoperative (μm)			
Coma	0.83 ± 0.32	0.74 ± 0.44	0.3734
Trefoil	0.23 ± 0.12	0.36 ± 0.24	0.1469
Spherical	2.24 ± 0.85	2.31 ± 0.50	0.8951

SE: spherical equivalent; TG: topography-guided; WFO: wavefront-optimized; LASIK: laser in situ keratomileusis. * Kruskal–Wallis test.

**Table 6:** Subjective quality of vision results obtained from questionnaire between TG-LASIK and WFO-LASIK groups after 3 months.

Parameter	TG-LASIK (20 Eyes)	WFO-LASIK (20 Eyes)	*p* Value *
Glare at night	36.3 ± 7.3	36.5 ± 6.4	0.3750
Haze	35.3 ± 6.7	37.8 ± 5.4	0.0215 *
Halos	36.4 ± 6.1	35.9 ± 4.4	0.6250
Clarity at night	34.6 ± 9.1	38.4 ± 6.4	0.0386 *
Clarity during the day	35.6 ± 7.2	37.4 ± 6.5	0.1094
Dry eye	36.2 ± 7.7	37.2 ± 7.7	0.3750
Dry eye, severity	36.4 ± 7.7	37.4 ± 7.7	0.3750
Fluctuating vision	35.8 ± 6.8	37.6 ± 6.8	0.2188
Total	35.8 ± 6.1	37.3 ± 4.6	0.0225 *

TG: topography-guided; WFO: wavefront-optimized; LASIK: laser in situ keratomileusis. * Wilcoxon signed rank test: *p* < 0.05.

## Data Availability

The data are available upon reasonable request to the corresponding author.
